# Cobalt (II) Ferrite-Based Organic–Inorganic Composite Material Formation and Its Properties

**DOI:** 10.3390/molecules31193482

**Published:** 2026-09-29

**Authors:** Alexander Vladimirovich Vyaltsev, Nina Petrovna Shabelskaya, Tatiana Vladimirovna Bauer, Elena Alexandrovna Yakovenko, Tatiana Mikhailovna Minkina, Asatullo Makhmadekubovich Radzhabov, Saglara Sergeevna Mandzhieva, Marina Alexandrovna Egorova, Oleg Alexandrovich Medennikov, Vera Andreevna Baranova

**Affiliations:** 1 Department of Ecology and Industrial Safety, Platov South-Russian State Polytechnic University (NPI), 346428 Novocherkassk, Russia; bgd-av@mail.ru (A.V.V.); yakovlena80@yandex.ru (E.A.Y.); rajabov.asadullo@mail.ru (A.M.R.); m.egorova@npi-tu.ru (M.A.E.); monomors@yandex.ru (O.A.M.); vera-npi@yandex.ru (V.A.B.); 2 Laboratory of Agrobiotechnology for Improving Soil Fertility and Agricultural Product Quality, Southern Federal University, 344006 Rostov-on-Don, Russia; bauer@sfedu.ru (T.V.B.); tminkina@mail.ru (T.M.M.); msaglara@mail.ru (S.S.M.)

**Keywords:** cobalt (II) ferrite, dye degradation, methyl orange

## Abstract

This paper examines the formation of cobalt (II) ferrite on the surface of an organic support in stages: mixing metal salts, interaction with ammonia and citric acid solutions, and the subsequent thermolysis of the intermediate products. The resulting materials were characterized by X-ray diffraction, electron microscopy, and elemental analysis. A comparative analysis of the materials’ morphological properties suggests the formation of a cobalt (II) ferrite film on the surface of the organic support. This film forms as small cubic crystals measuring 50–100 nm in size, with a simultaneous significant (2–5 times) increase in the specific surface area of the synthesized composite materials compared to the support surface. The typical pore diameter of the composites is 20–40 nm. The synthesized material of CoFe_2_O_4_/sunflower husk biochar exhibits high catalytic activity; dye is removed from the solution within 60 min. The material of CoFe_2_O_4_/sunflower husk ash is less active in this process: dye removal is observed 140 min after the start of the reaction. These data may be useful for developing catalytically active materials for wastewater and soil solution treatments to remove persistent pollutants.

## 1. Introduction

To obtain clean water, it is necessary to purify it. Removing unwanted impurities from water makes it possible to obtain purified water, which can be reused or discharged into a natural body of water. Low maximum permissible concentrations (MPCs) of dyes in water (from 0.0025 to 10 mg/dm^3^) and their complex aromatic molecular structures require the development of new wastewater treatment methods. Wastewater contaminated with dyes is generated during the production of finishing and decorative materials, in the textile and pharmaceutical industries. Wastewater in the presence of dyes, even at low concentrations, becomes intensely colored. Azo dyes pose the greatest toxicity risk to aquatic organisms, as they are resistant to biodegradation under aerobic conditions. Among currently available methods for wastewater treatment to remove organic matter, such as coagulation, flotation, ion exchange, membrane, and biological methods, two groups are of interest in terms of the use of porous inorganic materials: sorption and oxidation. Sorption methods are effective for the deep purification of water from dissolved organic compounds. However, there is a risk of secondary environmental contamination from the spent sorbent. In this regard, the most promising method is photocatalytic dye degradation [1,2,3].

Transition element ferrites are characterized by a set of properties that determine their wide application. They are chemically and thermally stable and possess magnetic properties [4,5,6]. In particular, cobalt (II) ferrite CoFe_2_O_4_ is used in biomedical applications for targeted drug delivery [1,7,8,9], as a material for absorbing electromagnetic radiation [10,11], as sensors for detecting drugs [12,13,14] and hydrogen [15], for storing and converting energy [16,17], and for magneto-optical device manufacturing [18]. Cobalt (II) ferrite is also an effective catalyst for organic dye degradation [3,19].

Methyl orange belongs to the group of azo dyes and is characterized by increased resistance to degradation. Table 1 provides information on the catalysts and their efficiency in methyl orange degradation.

The most common methods for synthesizing oxide photocatalysts are sol–gel [6,16,19,26,27], hydrothermal [8], and solid-phase [5] methods. The hydrothermal method requires specialized equipment, while the solid-phase method requires high temperatures; both methods are relatively time-consuming. The sol–gel method allows us to produce fine-crystalline materials under economical conditions.

In this regard, the aim of this study was to synthesize a composite material consisting of cobalt (II) ferrite on the surface of an organic support of sunflower husk ash and sunflower husk biochar and to use the synthesized material in the process of methyl orange dye removal from an aqueous solution.

## 2. Results

During the salt solution and ammonium hydroxide interaction, a precipitate was observed to form and then dissolve. After adding a citric acid solution, the precipitate dissolved, and the solution was reddish-brown.

An estimate of the ionic composition of the reaction mixture was performed.

In the system under consideration, the processes proceed in stages.

Stage 1: Mixing the salt solutions;

Stage 2: Interaction of Co(NO_3_)_2_ and Fe(NO_3_)_3_ salt solutions with ammonia NH_3_·H_2_O;

Stage 3: Introduction of citric acid C_6_H_8_O_7_ and formation of citrates.

Let us consider the processes occurring in the system at every stage.

Stage 1. Cobalt (II) and iron (III) nitrates undergo protolysis according to Equations (1)–(6). Metal nitrates are highly soluble in water, so the dissociation of the salts is irreversible (reactions 1 and 3). The hydrated cobalt cation in an aqueous solution acts as an acid, forming a hydroxocobalt (II) cation and an oxonium cation (reaction 2). The acidity constant (*K*_a_) is given after the reaction equation.

Co(NO_3_)_2_ = Co^2+^ + 2NO_3_^−^, (1)

Co^2+^·H_2_O + H_2_O ↔ CoOH^+^ + H_3_O^+^, *K*_a1_ = 6.3 · 10^−13^. (2)

Fe(NO_3_)_3_ = Fe^3+^ + 3NO_3_^−^, (3)

The iron (III) cation behaves similarly in an aqueous solution (reaction 4):

Fe^3+^·H_2_O + H_2_O ↔ FeOH^2+^ + H_3_O^+^, *K*_a2_ = 6.5 · 10^−3^. (4)

The hydroxyiron (III) cation formed during protolysis (reaction 4) can act as an ampholyte, exhibiting acidic (reaction 5) or basic (reaction 6) functions.

FeOH^2+^·H_2_O + H_2_O ↔ Fe(OH)_2_^+^ + H_3_O^+^, *K*_a3_ = 5.5 · 10^−4^, (5)

FeOH^2+^ + H_2_O ↔ Fe^3+^·H_2_O + OH^−^, *K*_b_ = 1.5 · 10^−12^. (6)

The acidity constant is significantly greater than the basicity constant. This suggests that FeOH^2+^ is more typically acidic, so Equation (6) was not considered in further calculations.

Based on Equations (2), (4) and (5), the equilibrium ion concentrations in the solution can be calculated (assuming that the initial salt concentrations were 1 mol/L):

- The following is for the process described by Equation (2):

*K*_a1_ = [CoOH^+^]·[H_3_O^+^]/[Co^2+^·H_2_O]; according to Equation (2), the concentrations of the reaction products are [CoOH^+^] = [H_3_O^+^], the concentration of the [Co^2+^·H_2_O] ion is equal to the concentration of the initial salt, and the concentration of oxonium ions [H_3_O^+^] = √*K*_a_ · C = √6.3 · 10^−13^ · 1 = 7.9 · 10^−7^ mol/L.

- For the process described by Equation (4), the reasoning is similar:

*K*_a2_ = [FeOH^2+^]·[H_3_O^+^]/[Fe^3+^·H_2_O]; the concentrations of the reaction products are [FeOH^2+^] = [H_3_O^+^], [H_3_O^+^] = 8.06 · 10^−2^ mol/L.

- Similarly, the following is for the process described by Equation (5):

*K*_a3_ = [Fe(OH)_2_^+^] · [H_3_O^+^]/[FeOH^2+^]; the concentrations of the reaction products are [Fe(OH)_2_^+^] = [H_3_O^+^], [H_3_O^+^] = 6.7 · 10^−3^ mol/L.

The total content of oxonium ions in the mixture of the initial salt solutions (due to the small quantity value in reaction (2), we neglect it) is 8.73 10^−2^ mol/L; therefore, the pH is 1.06.

Stage 2. When compounds in solution interact, ammine or hydroxy complexes may form (Table 2).

Table 2 shows that the most likely product of the interaction of metal salts with ammonium hydroxide is the formation of stable cobalt (II), iron (III) hydroxy complexes, and the corresponding hydroxides.

Table 3 shows the material balance of hydroxide anions in the solution.

The residual concentration of OH^−^ ions can be determined by the value of the solubility product constant (*K*_sp_) of cobalt (II) and iron (III) hydroxides:

*K*_sp Co(OH)2_ = 1.6 · 10^−15^, [OH^−^] = 2· (1.6 · 10^−15^/4)^1/3^ = 1.48 · 10^−5^ mol/L;

*K*_sp Fe(OH)3_ = 6.3 · 10^−38^. [OH^−^] = 3· (6.3 · 10^−38^/27)^1/4^ = 6.6 · 10^−10^ mol/L.

Consequently, the medium will be slightly alkaline, pH 9.1.

Stage 3. The solution contains the following compounds: Fe(OH)_3_, [Fe(OH)_4_], Co(OH)_2_, and H_3_O^+^.

Citric acid C_6_H_8_O_7_ is tribasic; the dissociation constants for the stages, according to reference data, are: *K*_ac1_ = 7.45 · 10^−4^, *K*_ac2_ = 1.73 · 10^−5^, and *K*_ac3_ = 4.09 · 10^−7^. During the dissociation of citric acid, the ions of C_6_H_7_O_7_^−^, C_6_H_6_O_7_^2−^, and C_6_H_5_O_7_^3−^ are formed. Since the acidity constant of citric acid for dissociation at the third stage is low, we assume that the compounds obtained during the dissociation of the acid in the first and, partially, in the second stage will participate in the formation of the product. Possible reactions can be described by Equations (7)–(11):

Fe(OH)_3_ + 3C_6_H_8_O_7_ = Fe(C_6_H_7_O_7_)_3_ + 3H_2_O, (7)

2Fe(OH)_3_ + 3C_6_H_8_O_7_ = Fe_2_(C_6_H_6_O_7_)_3_ + 6H_2_O, (8)

[Fe(OH)_4_]^−^ + 3C_6_H_8_O_7_ + H_3_O^+^ = Fe(C_6_H_7_O_7_)_3_ + 5H_2_O, (9)

Co(OH)_2_ + 2C_6_H_8_O_7_ = Co(C_6_H_7_O_7_)_2_ + 2H_2_O. (10)

Co(OH)_2_ + C_6_H_8_O_7_ = Co(C_6_H_6_O_7_) + 2H_2_O. (11)

The material balance for the amounts of citric acid is shown in Table 4.

Some of the oxonium ions formed in the initial stages are consumed in the reaction (9). Overall, after the formation of the intermediate synthesis products, the reaction system will be close to neutral.

## 3. Discussion

The resulting intermediate products were heated in a stainless-steel reaction vessel. Initially, the intense evaporation of the liquid was observed, resulting in the formation of a viscous gel-like precipitate. With further heating, the precipitate transformed into a solid porous product that was easily milled. This material then degraded, with abundant gas evolution. Localized heating to a red glow temperature was observed in certain parts of the system.

**Figure 1:**
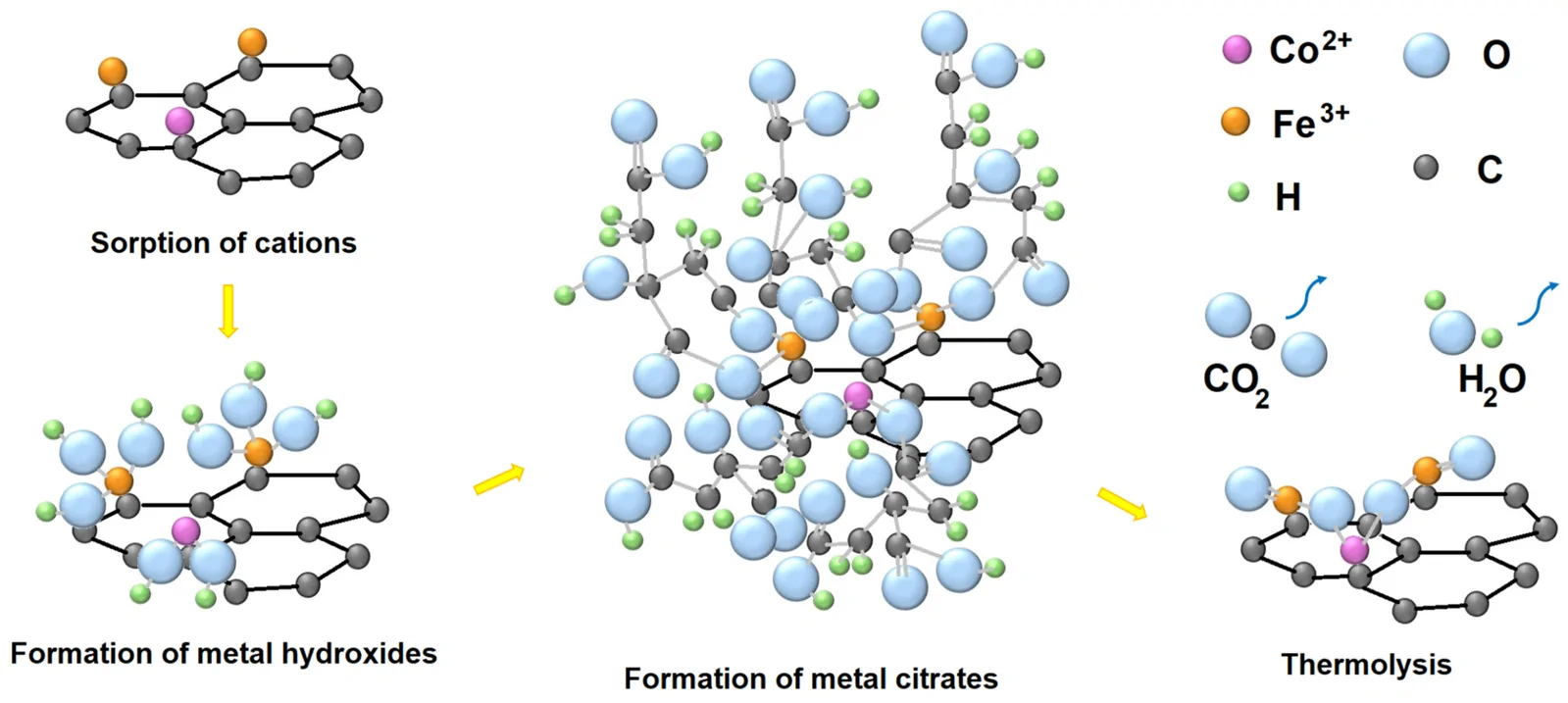
The formation of cobalt (II) ferrite on the surface of an organic support (yellow arrows indicate the transition between substances, blue arrows indicate the removal of products).

Figure 1 schematically shows the process of material formation.

**Figure 2:**
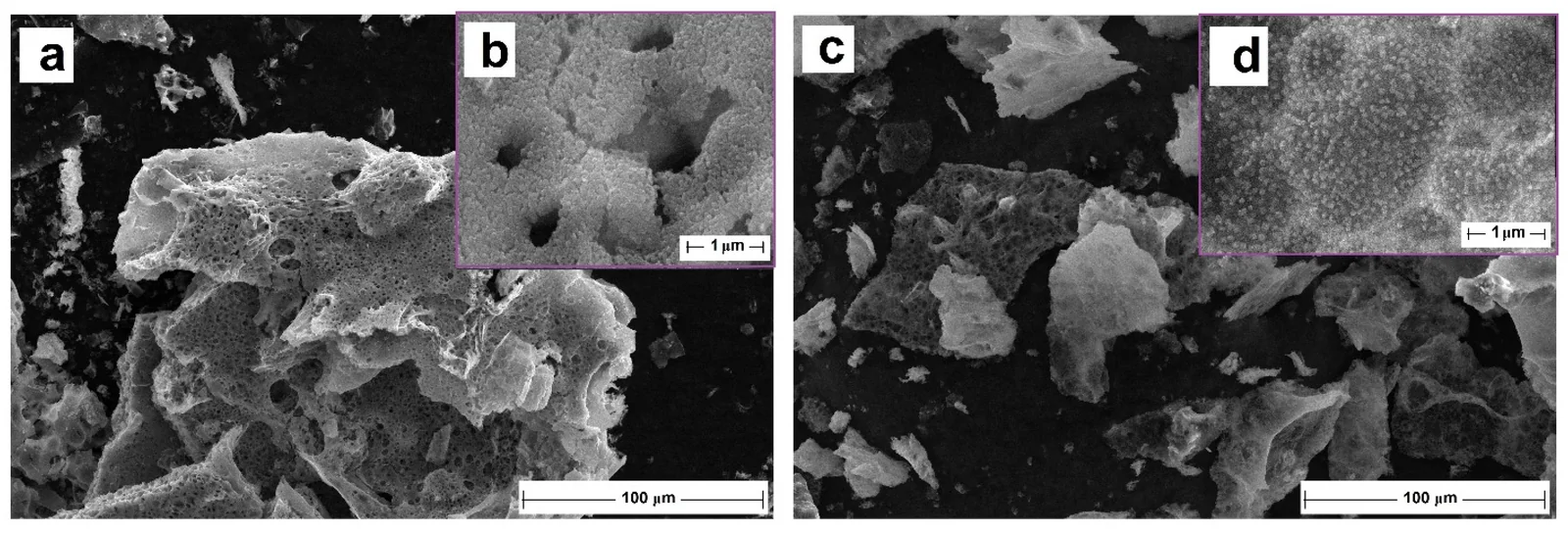
Micrographs of the composite material at various magnifications ((**a**,**c**)—×650; (**b**,**d**)—×50,000): (**a**,**b**)—CoFe_2_O_4_/sunflower husk ash; (**c**,**d**)—CoFe_2_O_4_/sunflower husk biochar.

After gas emission was completed, heating was stopped. The product was a highly porous material. Figure 2 shows micrographs of the resulting materials.

**Figure 3:**
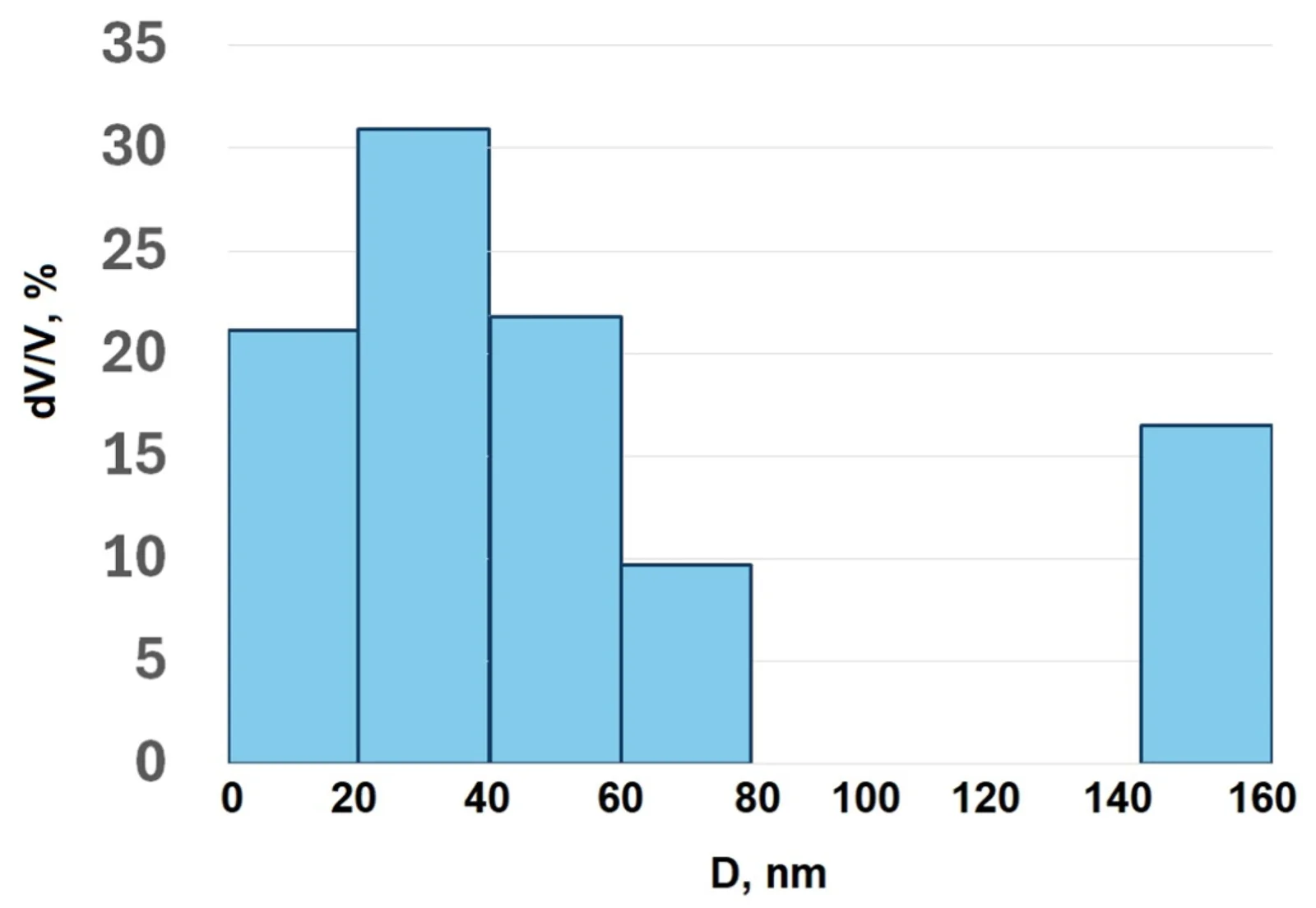
Distribution of pores in relation to their total volume for CoFe_2_O_4_/sunflower husk biochar sample.

**Figure 4:**
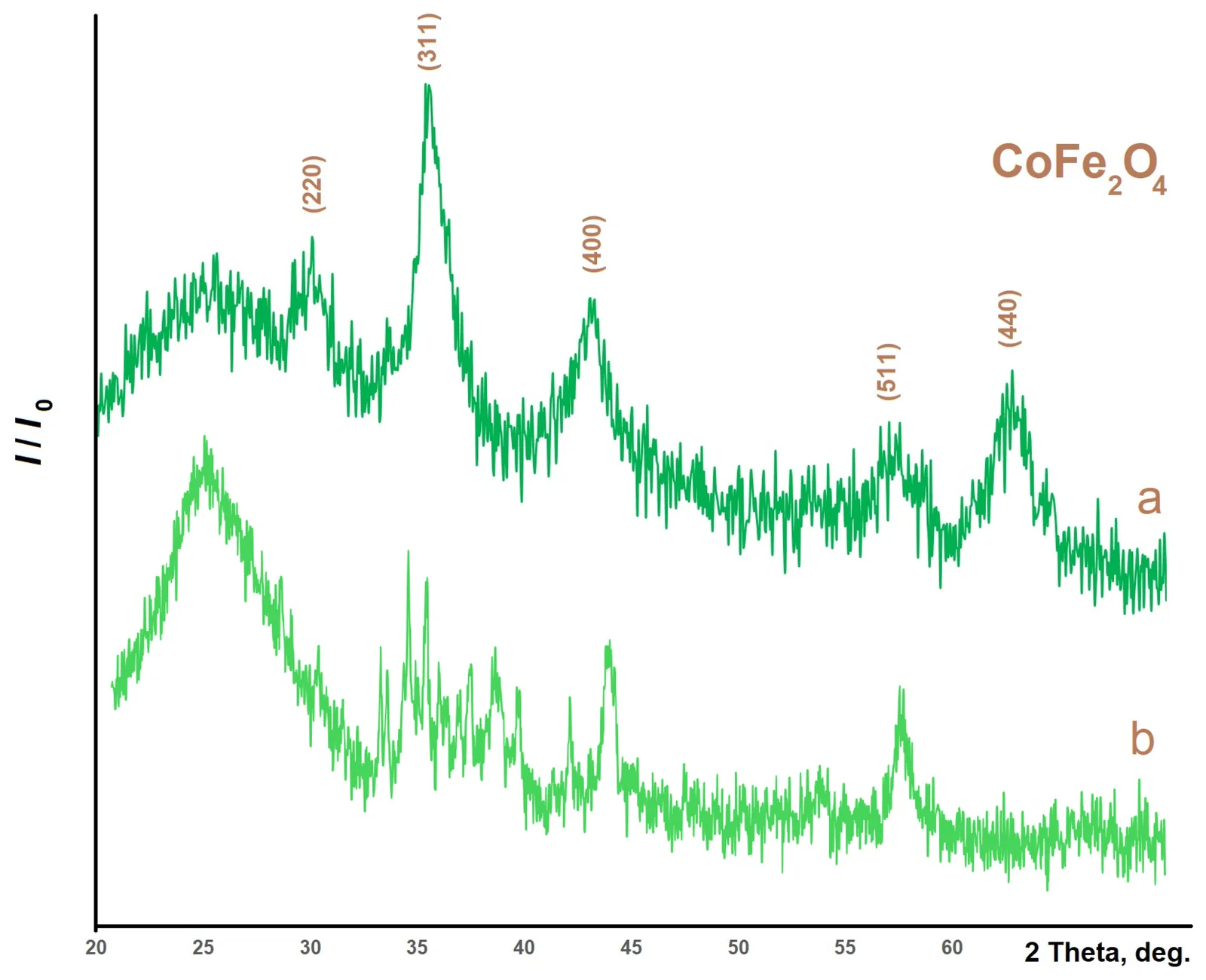
X-ray diffraction pattern of samples: (**a**)—CoFe_2_O_4_/sunflower husk biochar; (**b**)—CoFe_2_O_4_/sunflower husk ash.

**Figure 5:**
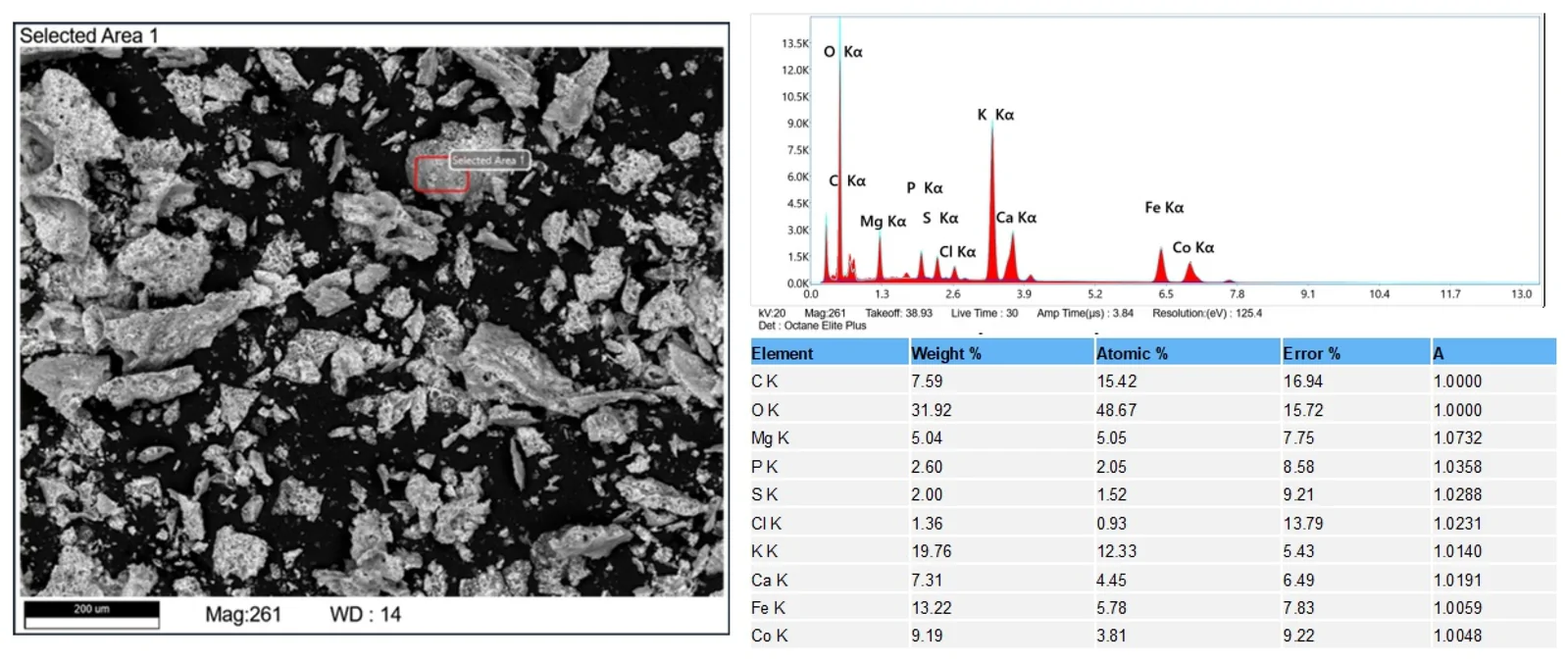
Elemental analysis data for the CoFe_2_O_4_/sunflower husk ash sample.

**Figure 6:**
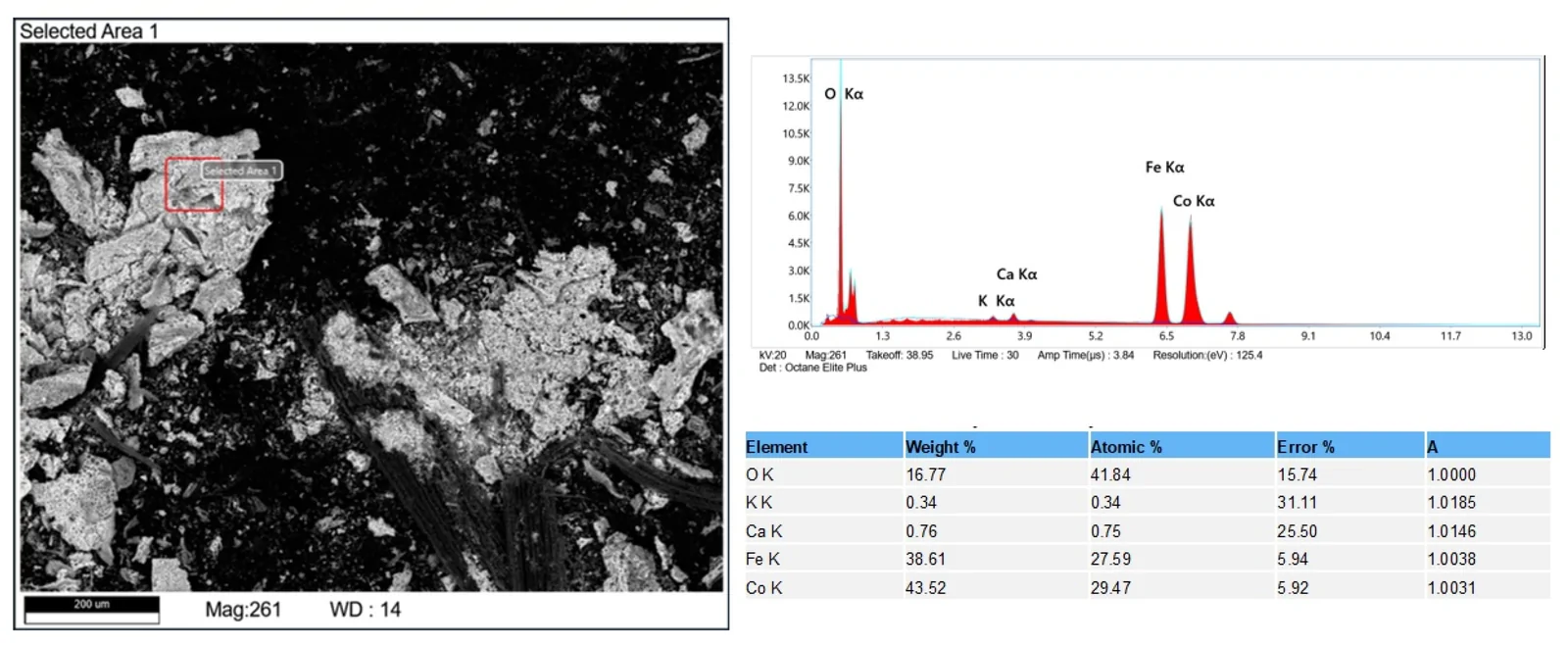
Elemental analysis data for the CoFe_2_O_4_/sunflower husk biochar sample.

**Figure 7:**
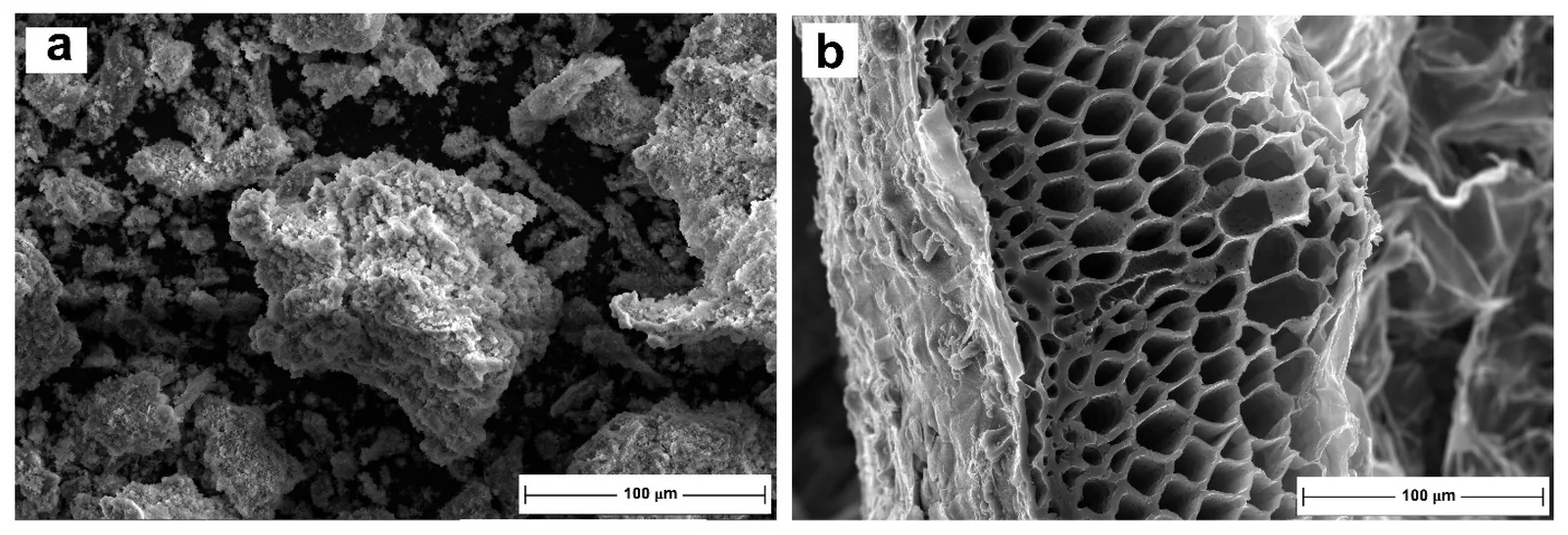
Micrograph of organic carrier: (**a**)—sunflower husk ash; (**b**)—sunflower husk biochar.

**Figure 8:**
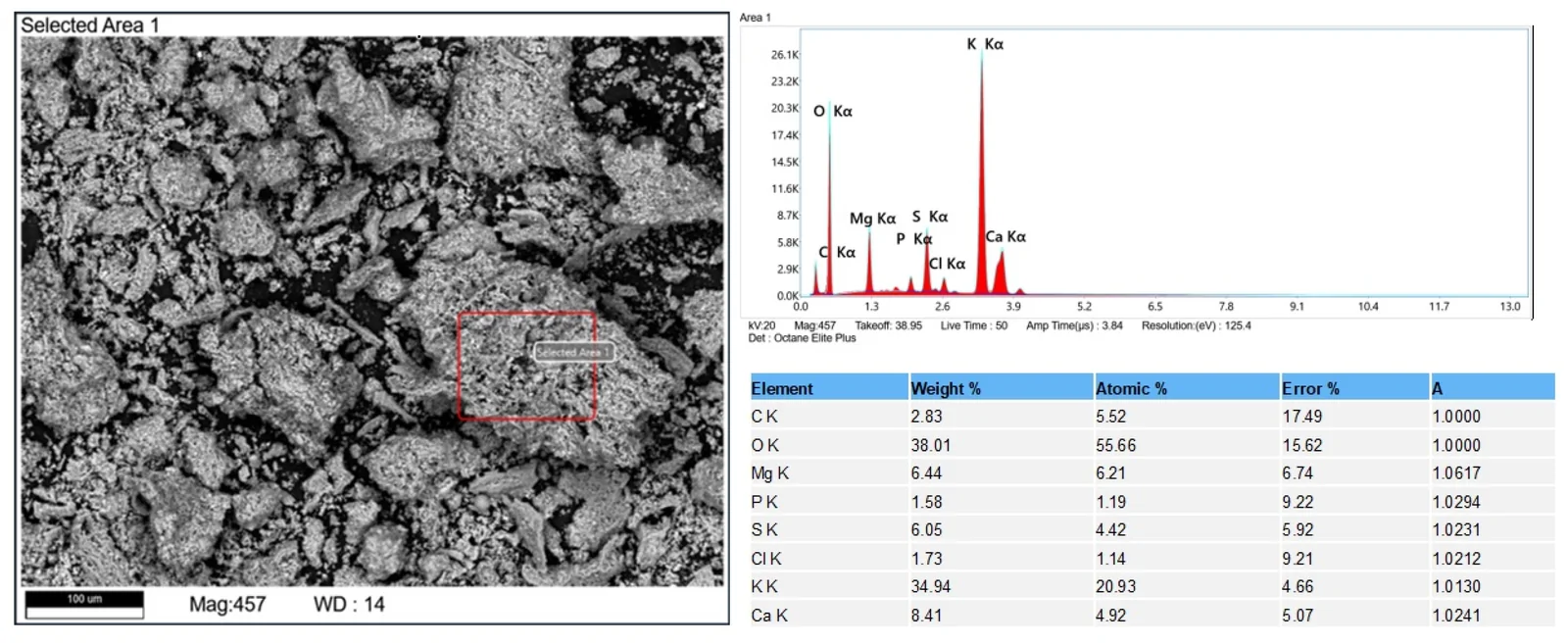
Elemental analysis data for the sunflower husk ash sample.

Figure 3, Figure 4, Figure 5, Figure 6, Figure 7 and Figure 8 provide a description of the materials. A comparative analysis of the materials (Figure 2a,c and Figure 7) suggests the formation of a cobalt (II) ferrite film on the surface of the organic support. The characteristics of the support and the composite are presented in Table 5.

Figure 2b,d show that cobalt (II) ferrite forms as small cubic crystals measuring 50–100 nm. They are likely responsible for the significant (2–5 times) increase in the specific surface area of the synthesized composite materials compared to the support surface. It can be assumed that the larger particles visible in the microphotograph are aggregates of crystallites. Figure 3 shows the pore distribution relative to their total volume for the sample with the most developed surface area (CoFe_2_O_4_/sunflower husk biochar). It is evident that the most characteristic pore diameter is in the range of 20–40 nm.

Figure 4 shows the X-ray diffraction pattern of the synthesized CoFe_2_O_4_/sunflower husk biochar composite. It reveals lines characteristic of cobalt (II) ferrite (PDF Number 010-74-6403); the biochar is X-ray amorphous. Cobalt (II) ferrite is characterized by peaks in the range of double angle values: 30.1, 35.5, 43.1, 57.0, and 62.6. The peaks in the X-ray diffraction pattern are significantly broadened, indicating a defective oxide phase. An estimate of the crystallite size using the Scherrer formula yields a value of 4.6 nm.

Figure 5 and Figure 6 show the elemental analysis data for the synthesized composite materials.

Figure 7 shows micrographs of the organic material used as a support for the composite systems.

**Figure 9:**
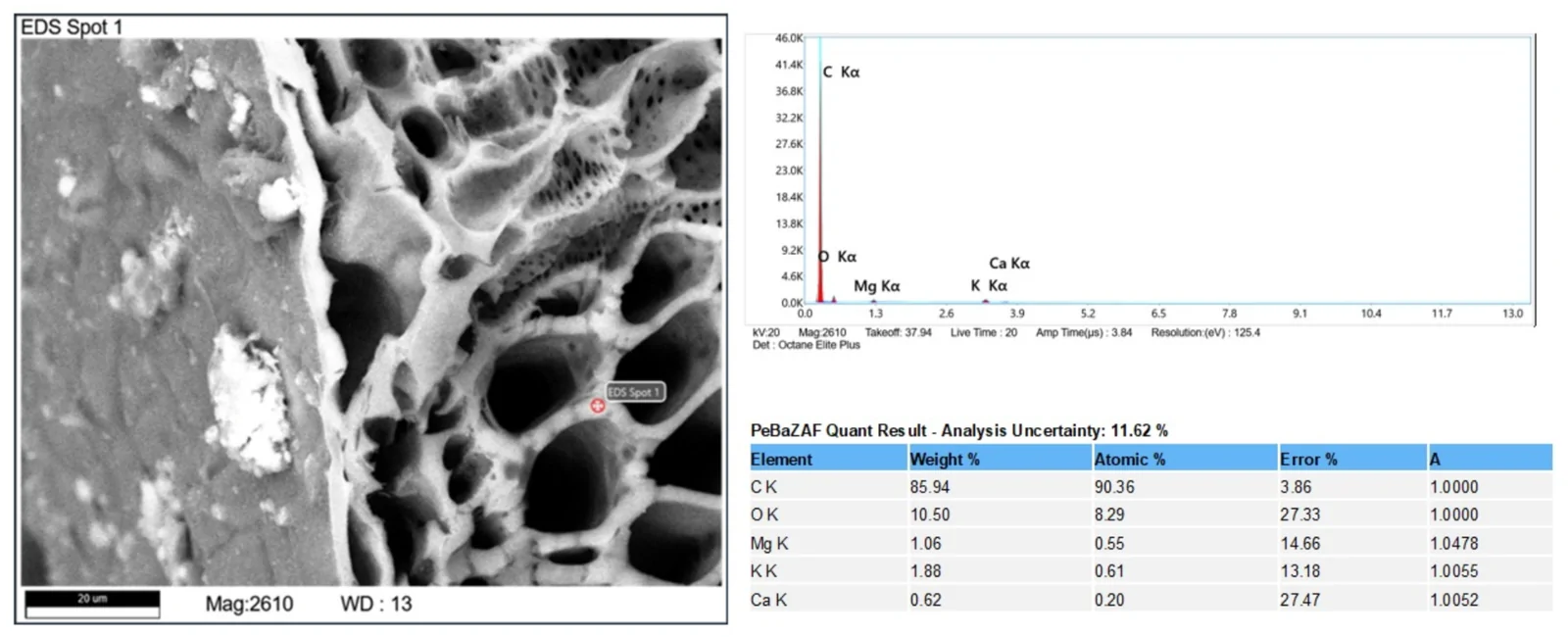
Elemental analysis data for the sunflower husk biochar sample.

Figure 8 and Figure 9 show the elemental analysis data for the organic materials used. Sunflower husk ash contains less atomic carbon than biochar.

A comparison of the morphological features and the data from the elemental analysis of the initial materials and composites allows us to conclude that the synthesis procedure was successfully completed: the data obtained indicate the successful formation of cobalt (II) ferrite on the support surface.

**Figure 10:**
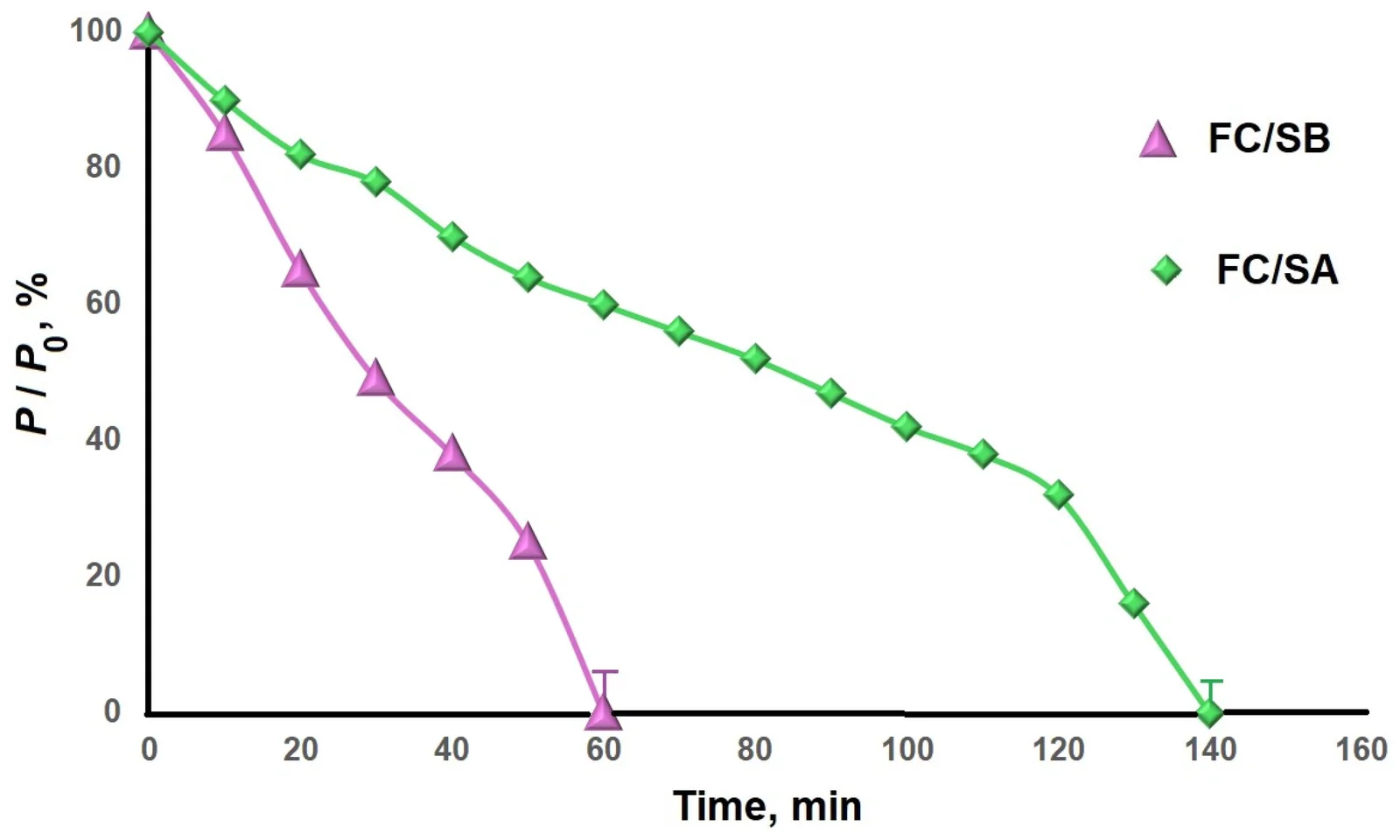
Dependence of methyl orange decomposition degree on reaction time.

The materials were tested in the degradation of organic dye (methyl orange) in an aqueous solution. Figure 10 shows the dependence of the amount of methyl orange on the reaction time. Samples containing organic support only were inactive in this process. A control measurement of the amount of methyl orange remaining in the solution after the adsorption equilibrium was established showed a value of 98–99%. Thus, it was concluded that the adsorption process does not play a significant role in the process of purifying an aqueous solution from methyl orange.

The synthesized CoFe_2_O_4_/sunflower husk biochar material exhibited high catalytic activity: in its presence, the dye was completely removed from the solution within 60 min. The CoFe_2_O_4_/sunflower husk ash material was less active in this process: dye removal was observed 140 min after the start of the reaction. This result may be due to the less developed surface area of the FC/SA material, which reduces the number of active sites.

The increase in catalytic activity may be due to the formation of a Fenton system during the redox process (Equations (12)–(21)):

CoFe_2_O_4_ + H_2_O_2_ → (CoFe^+2^Fe^+3^O_4_)^−^ + OH· + HO^+^, (12)

OH· + H^+^ → H_2_O, (13)

(CoFe^+2^Fe^+3^O_4_)^−^ + OH· → CoFe_2_O_4_ + OH^−^, (14)

OH^−^ + H^+^ → H_2_O, (15)

HO^+^ + OH^−^ → H_2_O + O^0^, (16)

H_2_O_2_ + O^0^ → H_2_O + O_2_, (17)

H_2_O_2_ → H^+^ + HO_2_^−^, (18)

H_2_O_2_ → 2OH^−^, (19)

HO_2_^−^ + HO^+^ → H_2_O + O_2_, (20)

O^0^ + (Methyl Orange) → (NOx + CO_2_ + H_2_O + …). (21)

A similar mechanism involving a highly reactive radical (OH) has been examined in several studies, for example, [21,28]. More complete dye degradation in an acidic environment may be due to the formation of an intermediate product based on cobalt (II) ferrite, which has a negative charge.

**Figure 11:**
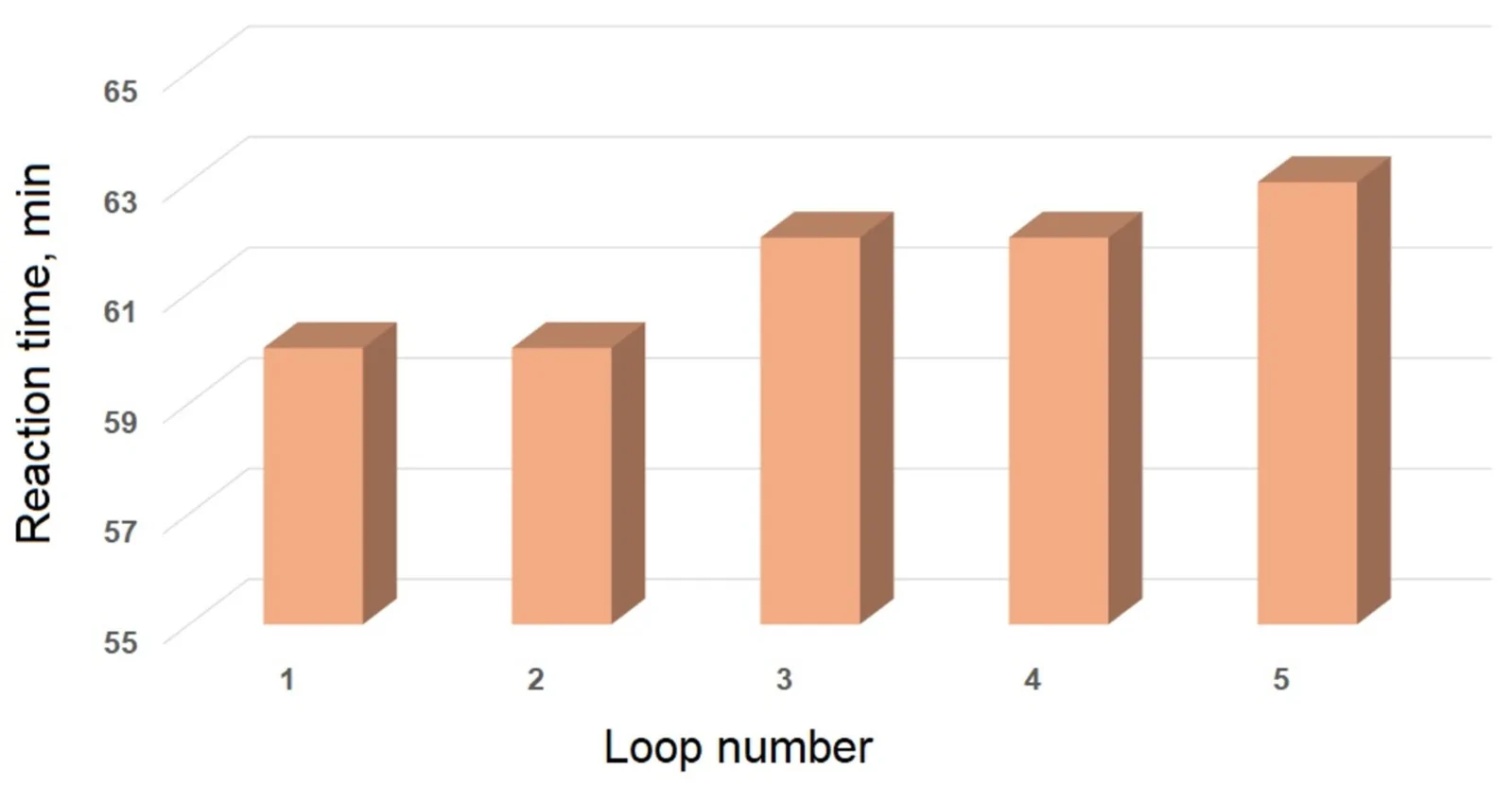
Reaction time for the decomposition of methyl orange depending on the cycle number.

The CoFe_2_O_4_/sunflower husk biochar catalyst was separated from the system by magnetic separation after the process was completed, washed three times with distilled water, and reused in the catalytic process. The catalyst can be used in five cycles without significant activity loss (Figure 11).

These data may be useful for developing catalytically active materials for wastewater and soil solution treatments to remove persistent pollutants.

## 4. Materials and Methods

### 4.1. Materials

The composite materials obtained in this study were based on an inorganic component of cobalt (II) ferrite and an organic component of sunflower husk ash and sunflower husk biochar. The obtained materials were synthesized in powder form using the sol–gel synthesis method. The following raw materials were used: aqueous ammonia (25% concentration) (JSC “Togliattiazot”, Tolyatti, Russia), citric acid monohydrate (C_6_H_8_O_7_·H_2_O) (“Russian Product”, Moscow, Russia), cobalt (II) nitrate hexahydrate (Co(NO)_3_)_2_ 6H_2_O) (JSC “LenReaktiv”, Saint Petersburg, Russia), and iron nitrate nonahydrate (Fe(NO)_3_)_3_ 9H_2_O) (JSC “LenReaktiv”, Saint Petersburg, Russia) of chemically pure grade.

### 4.2. Biochar Synthesis

The biochar synthesis is described in detail in [29]. Sunflower husk biomass was thoroughly washed with distilled water, dried until no further weight loss occurred, and subjected to stepwise pyrolysis in the absence of air at temperatures of 100–700 °C, with temperature increments of 200 °C, with intermediate temperature phases of 20 min and a final temperature phase of 45 min. The temperature increase rate was 11 °C/min. Sunflower husk ash was used in the form of fertilizer to reduce soil acidity.

### 4.3. Synthesis of Composite Materials

Before synthesis, solutions of transition element salts with a concentration of 1 mol/L and a citric acid solution with a concentration of 6.25 mol/L were prepared. The ammonia solution was used without dilution. Twenty-five grams of sunflower husk ash or sunflower husk biochar was measured into a reaction vessel and mixed with the transition element salt solution under continuous stirring. The salt solution was used in a ratio of Co(II):Fe(III) = 1:2, with 50 mL of iron (III) nitrate solution used. Ammonia (15 mL) and citric acid (25 mL) solutions were then added. The citric acid acted as a retention matrix, limiting particle growth and promoting the uniform dispersion of the nanosized ferrite particles.

The mixture was then heated. After the sol converted to gel, it ignited, ultimately forming a fine powder. The synthesis products were allowed to cool to room temperature.

The samples were subsequently designated as follows: sunflower husk ash—SA; sunflower husk biochar—SB; CoFe_2_O_4_/sunflower husk ash—FC/SA; CoFe_2_O_4_/sunflower husk biochar—FC/SB.

### 4.4. Material Characterization

Various methods were used to characterize the resulting composite materials, including X-ray diffraction (XRD) (Thermo Fisher Scientific, Ecublens, Switzerland), transmission electron microscopy, and the Scherrer method.

The phase composition was studied on an ARL X’TRA X-ray diffractometer (using monochromatic Cu-Kα radiation) using point-by-point scanning (0.01° step, 2 sec accumulation time per point) over a 2θ range from 20° to 70°. Crystallite size was calculated using the Scherrer equation [30]:

*D* = 0.94∙λ/(β∙cosθ),

where *D* is the average crystal size (nm), *λ* is the X-ray wavelength (nm), *β* is the peak linewidth at half maximum (rad), and *cos θ* is the cosine of the peak angle.

Morphology and chemical composition studies were performed using a Quattro S SEM (Thermo Fisher Scientific, Waltham, MA, USA) scanning electron microscope equipped with an Octane Elite Plus (EDAX, version 3.1) EDS microanalyzer.

Catalytic activity was studied using a model solution of methyl orange (λ_max_ = 465 nm), an organic dye. During the experiment, 10 mg of catalyst was weighed and placed in a reaction vessel. Then, 10 mL of a 0.05 g/L organic dye solution and 10 mL of a 3% hydrogen peroxide solution were added. Solutions of sulfuric acid and sodium hydroxide with a concentration of 1 mol/L were used to create a specific acidity. The dye in the solution was photocolorimetrically analyzed at specified time intervals using a KFK-2-UHL 4.2 instrument. A 100 W JC halogen lamp (Camelion, Camelion International Ltd., Shenzhen, China) was used as the light source. The distance from the light source to the reaction system surface was 50 mm. Before the experiment, the reaction system was thoroughly mixed, isolating it from light for 0.5 h to achieve adsorption/desorption equilibrium.

The degree of destruction P, %, was calculated using the following formula:

*P* = (*C*_0_ − *C*_t_) · 100/C_0_,

where *C*_0_ is the initial dye concentration in the solution, g/L, and *C_t_* is the amount of dye degraded at the current time, g/L.

## 5. Conclusions

The process of cobalt (II) ferrite formation on the surface of an organic support is examined in stages. It is shown that after mixing the initial salts, the total oxonium ion content is 8.73 × 10^−2^ mol/L; after the addition of an ammonia solution, hydroxides and hydroxy complexes are formed; after the addition of citric acid, citrates are formed.

The material formation process is presented. A comparative analysis of the morphological properties of the materials suggests the formation of a cobalt (II) ferrite film on the surface of the organic support. This film forms as small cubic crystals measuring 50–100 nm in size, with a simultaneous significant (2–5 times) increase in the specific surface area of the synthesized composite materials compared to the support surface. The most characteristic pore diameter of the composites was 20–40 nm.

The synthesized material of CoFe_2_O_4_/sunflower husk biochar exhibited high catalytic activity, completely removing the dye from the solution within 60 min. The material of CoFe_2_O_4_/sunflower husk ash was less active in this process: dye removal was observed 140 min after the start of the reaction.

These data may be useful for developing catalytically active materials for wastewater and soil solution treatments to remove persistent pollutants.

## Figures and Tables

**Table 1:** Catalyst composition and efficiency of methyl orange removal from aqueous solutions.

Catalyst	Purification Rate, %/Time, Min	Source
g-C_3_N_4_/Fe_3_O_4_@polyindole hybrids	97.5/70	[20]
Fe_50_%/Zn_50_-clinoptilolit	92.37/150	[21]
Ag NPs	100/150	[22]
Fe_3_O_4_-GO-TIO_2_	77.17/30	[23]
La_1−*x*_Gd*_x_*Mn_0.9_Zn_0.1_O_3_	97.0/60	[24]
Snowflake-rGO-CuO	87.0/80	[25]

**Table 2:** Stability constants (*K*_y_) of possible complex ions.

Formula	Stability Constant, *K*_s_	Formula	Stability Constant, *K*_s_
[Co^2+^(NH_3_)(H_2_O)_5_]	9.8 · 10^1^	[Co^2+^(OH^−^)_2_(H_2_O)_2_]	1.5 · 10^9^
[Co^2+^(NH_3_)_2_(H_2_O)_4_]	3.2 · 10^3^	[Co^2+^(OH^−^)_3_(H_2_O)]	3.1 · 10^10^
[Co^2+^(NH_3_)_3_(H_2_O)_3_]	2.7 · 10^4^	[Fe^3+^(OH^−^)(H_2_O)_3_]	6.8 · 10^11^
[Co^2+^(NH_3_)_4_(H_2_O)_2_]	1.2 · 10^5^	[Fe^3+^(OH^−^)_2_(H_2_O)_2_]	1.7 · 10^21^
[Co^2+^(NH_3_)_5_(H_2_O)]	1.4 · 10^5^	[Fe^3+^(OH^−^)_3_(H_2_O)]	7.4 · 10^32^
[Co^2+^(NH_3_)_6_]	2.5 · 10^4^	[Fe^3+^(OH^−^)_4_]	3.1 · 10^34^
[Co^2+^(OH^−^)(H_2_O)_3_]	2.5 · 10^4^		

**Table 3:** The material balance of hydroxide anions in the second stage of synthesis.

Income	Quantity, mol	%	Consumption	Quantity, mol	%
Introduced	6.7 · 10^−2^	100.0	Co^2+^ + 2OH^−^ = Co(OH)_2_	1.6 · 10^−2^	24.0
in the form of			CoOH^+^ + OH^−^ = Co(OH)_2_	1.9 · 10^−19^	0
NH_3_ · H_2_O			Fe^3+^ + 3OH^−^ = Fe(OH)_3_	4.18 · 10^−2^	62.9
			FeOH^2+^ + 2OH^−^ = Fe(OH)_3_	3.8 · 10^−3^	5.7
			Fe(OH)_2_^+^ + OH^−^ = Fe(OH)_3_	1.6 · 10^−4^	0.2
			Fe(OH)_3_ + OH^−^ = [Fe^3+^(OH^−^)_4_]	4.8 · 10^−3^	7.2
Deficiency	4.4 · 10^−4^	0.7	Total	6.656 · 10^−2^	100.0

**Table 4:** Material balance for the added citric acid.

Intake			Consumption		
Substance	Quantity, Mol	%	Substance	Quantity, Mol	%
Introduced			For formation		
C_6_H_8_O_7_	0.052	100.0	Co(C_6_H_7_O_7_)_2_	0.010	19.2
			Co(C_6_H_6_O_7_)	0.003	5.8
			Fe(C_6_H_7_O_7_)_3_	0.006	11.5
			Fe_2_(C_6_H_6_O_7_)_3_ from Fe(OH)_3_	0.009	17.3
			Fe_2_(C_6_H_6_O_7_)_3_ from [Fe(OH)_4_]^−^	0.024	46.2
Total	0.052	100.0	Total	0.052	100

**Table 5:** The characteristics of the support and the synthesized composite materials.

Substance	SA	SB	FC/SA	FC/SB
Surface area, *S*, m^2^/g	1.7 ± 0.0	25.9 ± 1.2	9.5 ± 0.2	55.1 ± 1.0

## Data Availability

Dataset available on request from the authors.
